# Effects of an Initial Muscle Strength Level on Sports Performance Changes in Collegiate Soccer Players

**DOI:** 10.3390/sports8090127

**Published:** 2020-09-15

**Authors:** Ai Ishida, Kyle Rochau, Kyle P. Findlay, Brandon Devero, Marco Duca, Michael H. Stone

**Affiliations:** 1 Center of Excellence for Sport Science and Coach Education, East Tennessee State University, Johnson City, TN 37614, USA; rochau@mail.etsu.edu (K.R.); KPFindlay@milligan.edu (K.P.F.); devero@mail.etsu.edu (B.D.); stonem@mail.etsu.edu (M.H.S.); 2 Dipartimento di Scienze Biomediche per la Salute, Università degli Studi di Milano, 20129 Milan, Italy; marco.duca@unimi.it

**Keywords:** collegiate sports, athlete monitoring, block periodization, specificity, strength training

## Abstract

The purposes of this study were to investigate effects of partial block periodized strength training on physical performance and to examine relationships between initial muscle strength measured with isometric mid-thigh pull (IMTP) and performance changes after 7 weeks of strength training. Seventeen collegiate male soccer players participated. Initial muscle strength was determined using IMTP while physical performance included 10 m and 20 m sprints and static vertical jump with a polyvinyl chloride pipe (SJ0), 20 kg barbell (SJ20), and barbell loaded to 40 kg bar (SJ40). Performance testing was performed at three points: before first week (baseline), fourth week (T1), and seventh week (T2). Statistically small to moderate changes were found from baseline to T2 in peak power (PP; *p* < 0.001, ES = 0.49), net impulse (NI; *p* < 0.001, ES = 0.49), peak velocity (PV; *p* < 0.001, ES = 0.62), allometrically scaled PP (PPa; *p* < 0.001, ES = 0.62) in SJ20 and jump height (JH) in SJ40 (*p* < 0.001, ES = 0.36). Moderate to large correlations were found between isometric peak force and the changes from baseline to T2 in SJ20 PP (*p* = 0.04, *r* = −0.49), SJ20 PF (*p* = 0.03, *r* = −0.52), PPa (*p* = 0.04, *r* = −0.50), and SJ20 allometrically scaled peak force (*p* = 0.04, *r* = −0.49). Properly structured strength training maximizes task-specific physical performance. Initial muscle strength negatively affects the magnitudes of adaptations to physical performance.

## 1. Introduction

Soccer is an intermittent physical activity consisting of sprinting, walking, jogging, jumping, kicking, heading, and changing directions. A soccer team consists of 11 players and is played on a field of 90–120 m long and 45–90 m wide. In collegiate soccer in North America, men play approximately 20 to 25 matches over 12 to 16 weeks. It is advantageous for collegiate players to have a high multifaceted physical capacity, such as relatively low body fat, high lean body mass (LBM), muscle strength, power, and aerobic endurance. Thus, improving physical capacity during the off-season is quite essential because recovery intervals and developmental time are limited between matches (2 to 4 days between matches) during the competitive season. However, off-season training to improve physical performance among collegiate soccer players can be quite limited because of collegiate regulations (e.g., 8 h of training sessions per week, and no supervised training during a spring break). Thus, sports scientists and coaches need to select an efficient periodization model to improve physical performance within limited time periods.

Block periodization is a model of process management that subdivides training into specific periods and fitness phases [1,2]. Single factor (single target) block periodization can be used to effect specific characteristics among athletes, including those in team sports, particularly in the off-season. One of the advantages of the model is to provide athletes sequential concentrated loads, each of which emphasizes one specific fitness character (i.e., strength-endurance, maximum strength, absolute strength, and strength-speed) and de-emphasizing other fitness characters in the phase. Continual unidirectional loads can potentiate the next fitness character and maximize sports performance at an appropriate time [1,3]. Some evidence suggest that athletes cannot maintain true peak performance more than about three weeks [1,3]. Thus, bringing an athlete to a peak using traditional methods can be quite difficult, especially if there are several important competitions close together. A potential advantage of block periodization is to allow for peak performance (to a point) to occur multiple times within a relatively short time span [3]. Therefore, there are good reasons why block periodization can be quite useful and make a profound difference in sports performance.

Maximum strength has been shown to be associated with increased power [4] and correlates with a number of factors related to sports performance such as sprinting and jumping [5,6]. Thus, there is good reason for athletes to increase maximum strength. However, evidence also indicates that the trained state can influence the outcome of gains on power and sport performance characteristics [6]. Athletes with higher initial muscle strength levels have been shown to gain maximum strength at a slower rate than weaker athletes [7]. Thus, it may be possible that initial muscle strength levels also influence the gains in power and sport-related physical performance tests resulting from strength training.

An athlete’s monitoring program has been used in competitive sports to evaluate athletes’ physical profile. The data from the monitoring program allow for maximizing athlete’s specific training-adaptations and physical preparedness at an appropriate time. For example, an athlete monitoring program can provide sports scientists and coaches with insights into how to prescribe training programs and/or manage fatigue based on the data.

Although a block periodization program improves sports performance in strength-power athletes [8], data are limited in collegiate male soccer players. Additionally, an athlete monitoring program can assess current athletes’ physical profile [9], yet it is still unclear as to the effect of initial maximum strength level on the changes in sports performance. Therefore, the purposes of this study were (a) to investigate the effects of a partial block periodized strength training program on sports-related physical performance (maximum strength, vertical jump, and sprint) and (b) to examine the relationships between initial muscle strength measured with the isometric mid-thigh pull (IMTP) and changes in sports-related physical performance in collegiate male soccer players in response to seven weeks of a strength training program.

## 2. Materials and Methods

### 2.1. Subjects

Seventeen National Association of Intercollegiate Athletics Colligate male soccer players were included for this study (19.6 ± 1.6 yrs; 73.8 ± 8.2 kg; 177.3 ± 5.6 cm). Players were excluded if (1) they could not complete all weight training sessions because of injuries and/or (2) they did not maintain accurate training logs in the weight training sessions. All players provided and signed a written informed consent prior to participation. This study was approved by the Institutional Review Board at East Tennessee State University on 29 April 2020 (c0420.3sdw).

### 2.2. Experimental Approach

This study used a descriptive observational design to monitor sports performance changes in relation to 7 weeks of strength training. The data collection for this study was routinely conducted as a part of an on-going athlete monitoring program. Initial muscle strength characteristics were determined by isometric peak force (IPF), rate of force development at 250 ms (RFD250), and net impulse to IPF (NIPF) using IMTP. As variables of interest relating to physical performances, data included performance aspects of the squat jump and sprinting. The variables were collected over a 7 weeks training program at three points: prior to the start of the first training block (baseline), the start of the second training block (T1), and the end of the second training block (T2). During the seven weeks of the data collection, players completed 2 to 4 training sessions per week on the practice field (session ratings of perceived exertion = 367.7 ± 228.2 arb. units) (Table 1). The strength training program was composed of 2 blocks of 3 weeks (strength-endurance and basic strength), followed by 1 week of a reduced training. In the reduced training week, the volume load was reduced by 30 to 40% from the previous week.

### 2.3. Training Program

In this study, a partial block periodization model was chosen, which consisted of two blocks (accumulation and transmutation) followed by 1 week of a reduced training (Table 2 and Table 3, respectively). Volume load (VL) was calculated by multiplying loads (kg) by reps by sets. Strength and conditioning coaches observed all the strength training sessions.

### 2.4. Measures

**Figure 1:**
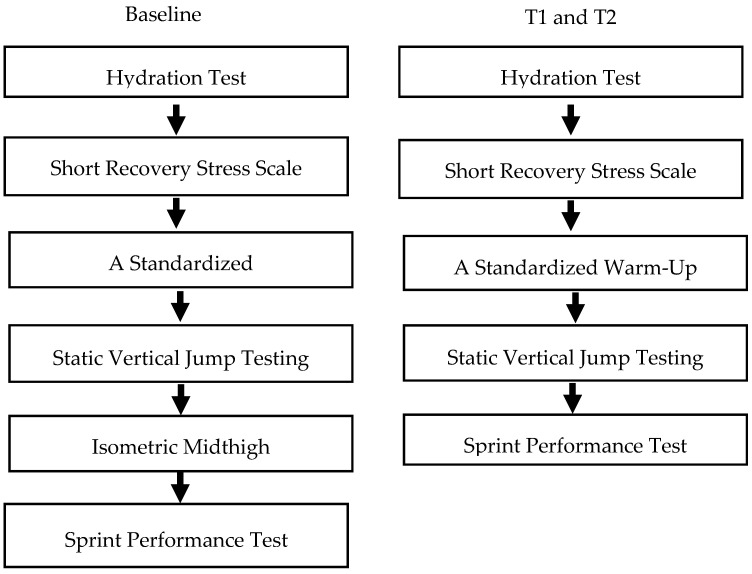
Testing schedule.

Short recovery stress scale (SRSS), jump performance, and sprint performance were assessed at baseline, T1 and T2 in laboratory and field settings. Static vertical jump and IMTP tests were performed in a laboratory setting while a 20-m sprint test was conducted on an artificial grass field. Testing procedure is shown in Figure 1. Before all the laboratory and field tests, hydration status was assessed using a refractometer (ATAGO, Tokyo, Japan). If urine specific gravity was ≤1.020, players were considered hydrated. If the USG was >1.020, athletes drank water until urine specific gravity indicated a hydrated state.

#### 2.4.1. Short Recovery Stress State 

Short recovery stress state (SRSS) was measured to quantify an athlete’s subjective recovery-stress state prior to jump testing. A customized online-based application (Google Forms, Google, Mountain View, CA, USA) was used to collect SRSS. SRSS includes 8 subscales such as physical performance capability (PPC), mental performance capability (MPC), emotional balance (EB), overall recovery (OR), muscular stress (MS), lack of activation (LA), negative emotional state (NES), and overall stress (OS). Players completed the SRSS immediately after the hydration test. Previous studies showed that SRSS has a good internal consistency in recovery and stress (α = 0.74 and α = 0.78) [10,11].

#### 2.4.2. Jump Performance 

Static vertical jump (SJ) was measured in a lab setting to evaluate neuromuscular performance. Participants performed a standardized warm-up consisting of 25 jumping jacks, and 1 set and 3 sets of 5 mid-thigh pulls with a 20 kg and 60 kg bar, respectively. SJ warm-up included a polyvinyl chloride pipe (SJ0), 20 kg barbell (SJ20), and barbell loaded to 40 kg (SJ40) bar at 50% and 75% of their perceived maximum efforts before making maximum efforts with each load. Each load was placed on their shoulders. Players were instructed to stand still on dual force plates (91.0 × 91.0 cm; Rice Lake Weighing Systems, Rice Lake, WI, USA), and maintain a squat position at a 90° knee angle measured with a goniometer. The athletes vertically jumped from the squat position without a countermovement on the command of “3, 2, 1, Jump”. If a tester visually observed evidence of a countermovement from the force-time curve, the trial was eliminated and the trial repeated. If a maximum effort trial differed by more than 2.0 cm the trial was repeated. Participants completed at least 2 trials for each load, and the mean of the best 2 trials in jump height (JH; cm) was analyzed using a customized LabVIEW program (2018 Version, National Instruments Co., Austin, TX, USA). JH derived from flight time, net impulse (NI; N), peak power (PP; W), peak force (PF; N), peak velocity (PV; m·s^−1^), allometrically scaled peak power (PPa; W·kg^−0.67^), and allometrically scaled peak force (N·kg^−0.67^) were calculated as the variables of interest. The coefficient of variation between the 2 trials in SJ JH, NI, PP, PFa, and PPa were 2.6% to 9.1%. Interclass correlation coefficient (ICC): 0.63 to 0.98). The ICC in PV ranges 0.49 to 0.69 with low coefficient of variation (CV) (6.8 to 8.4%).

#### 2.4.3. Isometric Mid-Thigh Pull Test

After the SJ test, IMTP testing was conducted on dual force plates (Rice Lake Systems, Rice Lake, WI, USA; 1000 Hz sampling rate) at baseline. Participants were instructed to flexed knee joints to 125 ± 5 degrees measured by a goniometer and to maintain an upright torso with extended elbows. An ITMP warm-up consists of 2 submaximal trials at 50% and 75% of their perceived maximal efforts. The participants pulled upward as fast and hard as possible on the commands of “3, 2, 1, Pull!”. Based on a visual force-time curve, if a tester observed a countermovement in the trial (<200 N), the trial was eliminated and repeated. Additional trials were performed if the IPF difference of the 2 IMTP was more than 200 N. Variables of interests relating to initial muscle strength levels were IPF (N), rate of force development at 250 milliseconds (RFD250; N·S^−1^), and net impulse to IPF (NIPF; N). In this study, CV between the 2 best ITMP were 5.0%, 23.3%, and 20.4% for IPF, RFD250, and NIPF. ICC for the variables were considered as moderate to excellent in this study (IPF: ICC = 0.94; RFD250: ICC = 0.78; NIPF: ICC = 0.60).

#### 2.4.4. Sprint Performance

A 20 m sprint test was performed (splits at 10 m and 20 m) using electronic wireless duel eye timing gates (Timing Ireland, Malahide, Dublin, Ireland). The height of the timing gates at the start and 10 and 20 m were at a knee joint and hip joint, respectively. Players stood with a staggered stance 30 cm behind from the start line. Prior to maximum trials, players completed standardized warm-up and 2 prints at 50 and 75% of their perceived maximum efforts. The players sprinted 2 times at their maximal efforts with 2–3 min rest between each sprint. The sprint trial was repeated if the athlete started poorly or slowed down prematurely. The fastest sprint times (s) in 10 m and 20 m sprints were used for data analysis. The CV for 10 m and 20 m ranges from 6.8 to 8.4%.

### 2.5. Statistical Analysis

The statistical software RStudio (version 1.1.463) with the packages dplyr (0.8.5), rstatix (0.4.0), and stats (3.5.3) was used to identify the relationship between initial muscle strength derived from IMTP (IPF, RFD250, and NIPF) and the changes in jump performances (JH, NI, PF, PP, PV) between three timings (baseline, Table 1 and Table 2). A Shapiro–Wilk test was performed to examine the normality of the data. A Friedman test and repeated measures analysis of variances were performed to compare the mean difference in SRSS and sports performance between baseline, T1, and T2, respectively. When necessary, a post-hoc test with a Bonferroni correction was conducted. Effect size (ES) values were classified as follows; ES < 0.2 = trivial, 0.2–0.6 = small, 0.6–1.2 = moderate, 1.2–2.0 = large, and >2.0 = very large [12]. Additionally, Pearson correlations were classified as trivial (0–0.09), small (0.10–0.29), moderate (0.30–0.49), large (0.50–0.69), very large (0.70–0.89), nearly perfect (0.90–0.99), and perfect (1.0) [12]. A Statistical significance for the analysis was set at *p* ≤ 0.05. All the data were expressed mean and standard deviation. 

## 3. Results

### 3.1. The Changes in Short Recovery Stress Scale

Table 4 describes the changes in short recovery stress scale between baseline, T1, and T2. No significant differences are observed between baseline, T1, and T2 in all scales (PPC: *p* = 0.17; MPC: *p* = 0.98; EB: *p* = 0.86; OS: *p* = 0.41; RS: *p* = 0.44; MS: *p* = 0.22; LA: *p* = 0.81; NES: *p* = 0.42; OS: *p* = 0.91; SS: *p* = 0.87).

### 3.2. Changes in Sports-Related Physical Performance

Table 5 summarizes the changes in sports performance. In SJ20, PP, NI and PV significantly small to moderately increased from baseline to T2 (PP: *p* < 0.001, ES = 0.49; NI: *p* < 0.001, ES = 0.49; PV: *p* = 0.003, ES = 0.83; PPa: *p* < 0.001, ES = 0.62). In addition, statistically significant small changes were noted between baseline and T2 in SJ40 JH (*p* < 0.001; ES = 0.36). Statistically significant increases were observed from T1 to T2 in SJ20 PP (*p* = 0.009, ES = 0.31), NI (NI: *p* = 0.003, ES = 0.31), and PF (*p* = 0.016, ES = 0.24) and SJ40 JH (*p* < 0.001, ES = 0.35). No statistically significant differences were seen between baseline, T1 and T2 in all SJ variables. The 10 m and 20 m sprint times at T2 were statistically lower than at T1 (10 m: *p* < 0.05, ES = 0.71; 20 m: *p* < 0.05, ES = 0.58).

### 3.3. Correlation between Initial Muscle Strength and Sports-Related Physical Performance

**Figure 2:**
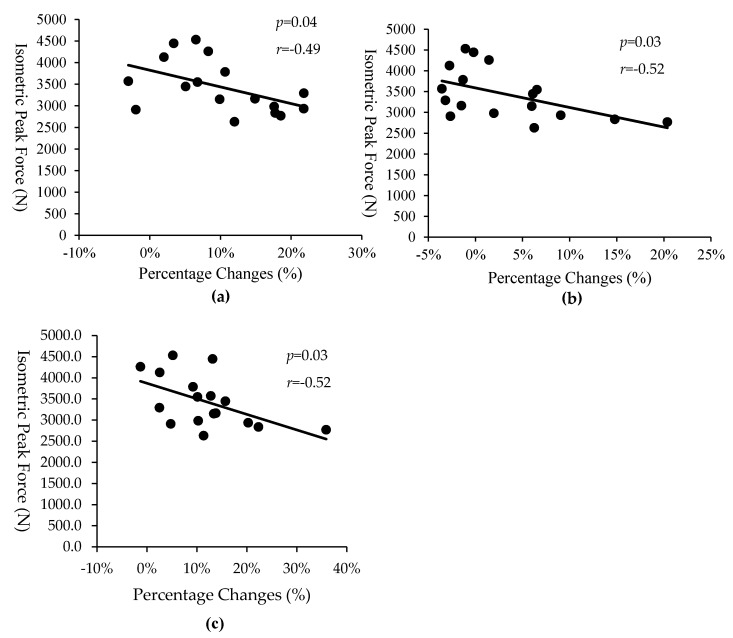
The correlation between isometric peak force and percentage changes from baseline to T2; the relationship with peak power in 20 kg static vertical jump (SJ) (**a**); the relationship with peak force in 20 kg SJ (**b**); the relationship with jump height in 40 kg SJ (**c**).

**Figure 3:**
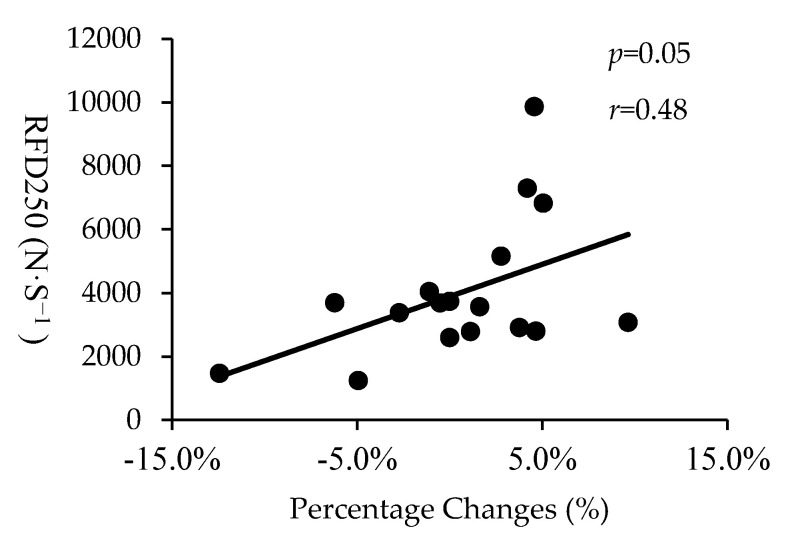
The correlation between rate of force development at 250 milliseconds (RFD250) and percentage changes from baseline to T1 in 10 m sprint time.

Table 6 describes the correlations between IPF, RFD250, and NIPF and the percentage changes of sports−related performance between baseline, T1 and T2. Moderate to large negative significant correlations were observed between IPF and the changes from baseline to T2 in SJ20 PP (*p* = 0.04, *r* = −0.49), SJ20 PF (*p* = 0.03, *r* = −0.52), SJ20 PPa (*p* = 0.04, *r* = −0.50) and SJ 20 PFa (*r* = −0.49, *p* = 0.04), and SJ40 JH (*p* = 0.03, *r* = −0.53) (Figure 2). NIPF was statistically significantly correlated to the changes of JH from baseline to T2 in SJ0 (*p* = 0.04, *r* = 0.50) and PF from T1 and T2 in SJ20 (*p* = 0.04, *r* = −0.51). However, no statistically significant correlations were noted for NI and PV at all loads. Initial RFD250 was significantly statistically correlated to the changes from baseline to T1 in 10 m sprint times (Figure 3). Negative correlations were found between NIPF and 20 m sprint times from baseline to T2 (*p* = 0.03, *r* = −0.54) and from T1 and T2 (*p* = 0.01, *r* = −0.59). No statistically significant correlations were observed between sprint performance and other jump variables between baseline, T1 and T2.

## 4. Discussion

A primary aim of this study was to investigate the relationships between initial muscle strength level and the changes in sports−related physical performance after seven weeks of strength training in collegiate male soccer players. This was the first study to identify the relationships between initial muscle strength from IMTP and physical performance changes in competitive collegiate soccer players. The main findings of this study were (a) PP, NI, and PV in SJ20 and JH in SJ40 at T2 were statistically higher than at baseline, (b) negative moderate to large correlations were found between initial IPF and the changes from baseline to T2 in SJ20 PP, SJ20 PF, and SJ40 JH, and (c) no statistically significant correlations were observed between baseline and T1 in jump performance.

NI, PP, PV, and PPa in SJ20, and JH in SJ40 statistically improved from baseline to T2. Importantly, properly structured strength training would improve average force production capacity (NI) with increased velocity (PV) in loaded conditions, leading to increasing PP and PPa. Current evidence [6,7,13] indicates that neuromuscular changes (i.e., motor unit rate coding, neural drive, and inter− or intra−muscular coordination) would result in performance alterations. The statistically significant changes in PPa could exclude the effects of morphological changes (i.e., LBM, and muscle cross-sectional area: mCSA) because power output per body was improved increased [7,13]. This agrees with the findings of Ahtiainen and his colleagues [7] who found that the relative maximal peak force in knee extension was statistically increased in untrained men and trained athletes after to 21 weeks of strength training (both *p* < 0.01). Therefore, in this group of athletes, neural changes would appear to predominate in response to seven−week of structured strength training. However, this finding does not preclude the possibility of alterations in specific fiber type cross-sectional area (CSA) or the II:I CSA area ratio that could have influenced performance [14].

In this study, initial IPF was negatively correlated to the changes from baseline to T2 in SJ20 PP, SJ20 PF, SJ20 PPa, SJ20 PFa, and SJ40 JH. This study indicates that initial muscle strength level may affect the magnitude of PF, PP, PPa, and PFa in loaded jump performances. Similar to the findings of this study, Ahtiainen et al. [7] found that a larger change was observed in the ratio of knee extension 1RM to mCSA of quadriceps in physically active men (10.6%) compared to strength athletes (8.2%) after 21 weeks strength training. Based on the findings of this study and the evidence of previous literature [7], superior neuromuscular adaptations to strength training seems to occur in weaker athletes compared to stronger athletes. However, the results of some studies indicated that initial muscle strength level would not affect the improvements in sports performance in response to strength and power training [4,15]. Cormie, McGuigan, and Newton [4] reported that both stronger and weaker muscle strength groups significantly improved jump squat and 40 m sprint performances after ten weeks of power training. One possible explanation of the disagreement may be the training regimen. In this study, a partial block periodization program was used to emphasize maximum strength gains, while Cormie, McGuigan, and Newton [4] chose 12 weeks of power training. It is possible that expected adaptations to strength training may not occur if training is not is properly structured to achieve given fitness goals in each training block (i.e., increasing work capacity and changing body composition for the strength−endurance phase and increasing maximal strength and power capacity for the muscle strength phase, respectively). Interestingly, Cormie et al. [16] indicated that among relatively weak subjects, strength training alone produced improvements in power and movement velocity as good or better than power training, a finding consistent with the results of the present study. 

Interestingly, positive correlations were found between initial IPF and PV between baseline and T1 or T2 although the relationships were not statistically significant. These findings suggest that large adaptations to strength training could occur in PV in stronger athletes compared to weaker athletes. It is well-known that RFD, PP, and PV can be substantially affected by muscle strength [17,18,19,20], so stronger athletes can produce a higher force at higher velocity with a given load compared to weaker athletes. According to Stone et al. [20], the force−velocity curve can shift toward the high-velocity end when power training is emphasized. For instance, PP and PV of SJ0 in untrained men statistically increased by 12.5 W·kg^−1^ and 0.52 m·s^−1^ after 12 weeks of lower−body power training [18]. Evidence also suggests that both strength and power may be enhanced markedly using combination training that includes heavy and light days [21,22]. In this current study, as a result of heavy and light days, movement velocity was especially emphasized (i.e., move the weights faster) on Day 3 of the strength-endurance and the strength phases. Thus, the training program in the current study may have affected the association between initial IPF and PV.

Importantly, no statistically significant changes were observed in SJ0 between baseline and T1 or T2. Additionally, IPF and RFD250 were not significantly statistically correlated to the changes in SJ0 variables from baseline to T1 or T2. The findings also suggest that performance changes and correlations would exhibit a degree of task-specificity to loads applied. Evidence indicates that task−specificity is an important factor to influence the magnitude of performance changes [2,3]. The mechanical specificity of a task is a primary mechanism determining the transfer of training effects to performance changes [23]. Force output and movement velocity of SJ0 appear to be less affected by the programming used compared to SJ20 and SJ40. For example, concentric PF increased with increased external loads in countermovement jump (*p* < 0.05) while PV decreased (*p* < 0.05). Based on the findings of this study and previous literature [15,18,22,23], the transfer of training effects for SJ0 was lower than SJ20 and SJ40 as might be expected without a shift of training emphasis from strength to higher velocities and explosiveness. This agrees with previous findings of McBride et al. [24] who found that performance changes in un−load and loaded squat jumps are sensitive alterations resulting from training with various loads in strength−power training. Therefore, strength−speed emphasis training should be incorporated to maximize the transfer of training effects on sports performance.

A statistically significantly moderate positive correlation was observed between initial RFD250 and the changes from baseline to T1 in 10m sprint time. RFD is defined as the ability to produce forces at a given time during a rapid voluntary contraction and commonly measured to assess explosive strength [25]. In this current study, weaker athletes positively changed 10 m sprints performance after the strength−endurance phase, while the sprint time in stronger athletes increased. One possible explanation for the changes may be neural adaptation to strength training. It is noted that adaptive neural alterations to strength training have been observed after 4 weeks of strength training [13,25]. For example, Staron et al. [13] showed that healthy men significantly increase 1RM leg press without morphological changes after 4 weeks of resistance training. Similar to previous literature [13,25], the weaker athletes in this study may have developed superior neural adaptations to strength training, leading to reducing 10m sprint times. 

There are two primary limitations in this study. First, morphological changes (i.e., LBM and mCSA) and maximal strength (i.e., IPF and RFD) were not assessed. This information would allow researchers to better understand what factors underlie performance changes. Second, in this current study, researchers did not prescribe the training volume and intensity in the training sessions. Although no statistically significant differences were found in SRSS between baseline, T1, and T2, residual fatigue from the training sessions might have affected the testing performances in T1 and T2. Future studies should include morphological markers such as LBM or mCSA to quantify the effects of the changes on sports performance improvement. 

## 5. Conclusions

The results of this study indicate that a structured, sequenced strength training program, emphasizing strength-endurance and maximum strength, can increase task-specific variables associated with sports performance. Additionally, the initial muscle strength level may negatively impact the magnitudes of adaptations to sports performances in response to strength training.

## Figures and Tables

**Table 1:** Overall Training and Testing Schedule for 7 Weeks.

Week	Mon	Tue	Wed	Thu	Fri	Sat	Sun
1	Wt	OFF	Wt	OFF	Wt	OFF	OFF
2, 3	Tr, Wt	Tr	Tr, Wt	Tr	Tr, Wt	OFF	OFF
4	T, Tr, Wt	Tr	Tr, Wt	Tr	Tr, Wt	OFF	OFF
5, 6	Tr, Wt	Tr	Tr, Wt	Tr	Tr, Wt	Match	OFF
7	Wt	Tr	Wt	Tr	T, Wt	OFF	OFF

Note. Wt = weight training. Tr = soccer-related training sessions. T = testing.

**Table 2:** Training Program for 7 Weeks.

Phase	Week	Sets and Reps	Daily Relative Intensities (D1, D2, D3)	Volume Load (kg)
SE	1	3 × 10 (3 × 5)	ML, ML, VL	17,221 ± 2548
SE	2	3 × 10 (3 × 5)	M, M, L	18,800 ± 2735
SE	3	3 × 10 (3 × 5)	H, H, L	19,992 ± 2966
MS	4	5 × 5 (5 × 3)	ML, ML, VL	16,333 ± 2145
MS	5	3 × 5 (3 × 3)	M, M, L	12,159 ± 1486
MS	6	3 × 5 (3 × 3)	H, H, L	12,664 ± 1670
MS	7	3 × 3	VL, VL, VL	8053 ± 1595

**Note.** D1 = Day 1. D2 = Day 2, D3 = Day 3. SE = strength-endurance. MS = muscle strength. VL = very light (65–70%). L = light (70–75%). ML = moderate light (75–80%), M = moderate (80–85%). MH = moderate heavy (85–90%). H = heavy (90–95%).

**Table 3:** Exercise selection for 7 weeks.

Day	Strength-Endurance	Muscle Strength
Day 1 and Day 3	Back Squat	Back Squat
	BB Shoulder Press	BB CG Push Press
	DB Lunge	DB Step Up
	BB Bench Press	BB Bench Press
	DB Triceps Extension	
Day 2	Clean Pull	Pull from Knee
	Straight Leg Deadlift	Straight Leg Deadlift
	Bent Over Row	Bent Over Row
	DB Pull Over	DB Pull Over
	DB Biceps Curl	

Note. BB = barbell. CG = clean grip. DB = dumbbell.

**Table 4:** The changes of short recovery stress scale across three timings.

Variables	Baseline	T1	T2
Physical Performance Capability	4.6 ± 0.9	5.1 ± 0.7	4.9 ± 0.7
Mental Performance Capability	4.8 ± 1.0	5.2 ± 0.9	5.1 ± 1.1
Emotional Balance	5.4 ± 0.5	5.4 ± 0.9	5.1 ± 0.9
Overall Recovery	5.1 ± 0.6	5.1 ± 0.8	4.8 ± 0.7
Recovery Scale	5.0 ± 0.5	5.2 ± 0.7	5.0 ± 0.7
Muscle Stress	1.8 ± 1.4	0.9 ± 0.6	1.1 ± 0.9
Lack of Activation	1.0 ± 1.1	0.9 ± 0.8	1.0 ± 0.9
Negative Emotional State	0.8 ± 1.2	0.70 ± 1.3	0.9 ± 1.1
Overall Stress	1.2 ± 1.5	0.9 ± 0.7	1.1 ± 1.1
Stress Scale	1.2 ± 1.0	0.9 ± 0.7	1.0 ± 0.9

Note. Values are expressed as means ± SD. T1 = the start of the second training block. T2 = the end of the second training block (T2).

**Table 5:** Changes in physical performance across three timings.

	Time Points			Percent Changes (ES)		
Variables	B	T1	T2	B-T1	B-T2	T1-2
SJ 0 kg						
JH (cm)	32.4 ± 6.7	32.3 ± 6.6	32.9 ± 6.7	−0.2 ± 7.2 (−0.02)	1.6 ± 5.9 (0.08)	2.1 ± 11.8 (0.09)
NI (N)	195.6 ± 28.7	193.4 ± 30.7	199.0 ± 31.7	−1.1 ± 6.9 (−0.08)	2.1 ± 9.9 (0.11)	3.1 ± 6.4 (0.18)
PP (W)	4126.2 ± 810.7	4055.9 ± 883.1	4276.5 ± 902.3	−1.6 ± 9.0 (−0.08)	4.4 ± 13.2 (0.18)	6.0 ± 9.3 (0.25)
PF (N)	1698.3 ± 252.7	1697.7 ± 257.3	1738.2 ± 215.6	0.1 ± 4.3 (0.00)	3.0 ± 7.3 (0.17)	2.9 ± 5.7 (0.17)
PV (m·s^−1^)	2.87 ± 0.26	2.81 ± 0.30	2.93 ± 0.37	−1.8 ± 8.2 (−0.21)	2.3 ± 10.6 (0.19)	4.2 ± 7.6 (0.35)
PPa (W·kg^−0.67^)	232.1 ± 38.0	226.6 ± 44.4	241.9 ± 48.5	−2.2 ± 10.1 (−0.13)	4.8 ± 13.9 (0.23)	**7.1 ± 10.3 (0.33)**
PFa (N·kg^−0.67^)	95.5 ± 9.2	94.8 ± 9.5	98.2 ± 8.0	−0.7 ± 4.3 (−0.07)	3.4 ± 8.7 (0.32)	**4.0 ± 7.0 (0.39)**
SJ 20 kg						
JH (cm)	23.5 ± 5.5	23.8 ± 5.3	24.4 ± 4.7	2.1 ± 11.8 (0.06)	5.1 ± 8.1 (0.19)	3.8 ± 9.6 (0.13)
NI (N)	206.8 ± 30.4	211.7 ± 32.4	221.3 ± 28.6	2.5 ± 7.3 (0.16)	**7.4 ± 5.9 (0.49)**	**5.0 ± 5.2 (0.31)**
PP (W)	3895.4 ± 768.3	4023.6 ± 793.0	4255.8 ± 704.4	3.6 ± 9.2 (0.16)	**10.1 ± 7.8 (0.49)**	**6.8 ± 8.5 (0.31)**
PF (N)	1833.9 ± 249.4	1830.4 ± 243.5	1885.7 ± 212.1	0.0 ± 5.3 (−0.01)	3.3 ± 6.6 (0.22)	**3.3 ± 4.1 (0.24)**
PV (m·s^−1^)	2.43 ± 0.20	2.55 ± 0.33	2.61 ± 0.22	4.8 ± 10.1 (0.44)	**7.3 ± 6.4 (0.83)**	2.9 ± 6.8 (0.20)
PPa (W·kg^−0.67^)	186.1 ± 31.1	197.3 ± 39.1	204.7 ± 29.0	6.0 ± 10.2 (0.32)	**10.7 ± 8.1 (0.62)**	5.0 ± 8.5 (0.22)
PFa (N·kg^−0.67^)	87.7 ± 8.2	88.9 ± 7.7	90.7 ± 5.9	1.5 ± 3.7 (0.15)	3.7 ± 5.7 (0.42)	2.2 ± 3.5 (0.26)
SJ 40 kg						
JH (cm)	16.0 ± 4.9	16.1 ± 4.4	17.7 ± 4.6	1.9 ± 10.8 (0.02)	**11.9 ± 8.8 (0.36)**	**10.5 ± 9.3 (0.35)**
NI (N)	214.3 ± 33.2	225.1 ± 43.5	226.1 ± 41.9	5.0 ± 11.5 (0.28)	5.6 ± 11.8 (0.31)	1.1 ± 10.4 (0.02)
PP (W)	3790.5 ± 713.3	3994.4 ± 934.5	4050.0 ± 872.0	5.3 ± 13.1 (0.25)	7.0 ± 12.2 (0.33)	2.4 ± 11.7 (0.06)
PF (N)	2002.1 ± 245.6	2012.3 ± 232.4	2051.2 ± 218.7	0.7 ± 3.8 (0.04)	2.8 ± 5.4 (0.21)	2.1 ± 3.1 (0.17)
PV (m·s^−1^)	2.14 ± 0.20	2.24 ± 0.33	2.25 ± 0.32	4.6 ± 12.1 (0.37)	4.8 ± 12.1 (0.39)	0.8 ± 11.0 (0.02)
PPa (W·kg^−0.67^)	159.0 ± 25.8	167.8 ± 37.4	170.3 ± 34.5	5.5 ± 14.7 (0.22)	7.2 ± 13.7 (0.37)	2.7 ± 13.5 (0.07)
PFa (N·kg^−0.67^)	84.1 ± 7.0	84.67.1	86.2 ± 6.0	0.7 ± 4.0 (0.07)	2.7 ± 4.2 (0.33)	2.1 ± 3.5 (0.25)
Sprint						
10 m Time (s)	1.81 ± 0.09	1.81 ± 0.05	1.78 ± 0.06	0.7 ± 3.2 (0.03)	−0.9 ± 3.3 (0.48)	**−1.6 ± 2.6 (0.71)**
20 m Time (s)	3.08 ± 0.12	3.10 ± 0.08	3.05 ± 0.09	0.4 ± 5.2 (0.18)	−1.8 ± 5.4 (0.29)	**−2.1 ± 3.8 (0.58)**

Note: Values are expressed as means ± SD (effect size). ES = effect size. B = baseline. T1 = the start of the second training block. T2 = the end of the second training block. SJ = static vertical jump. JH = jump height. PP = peak power. NI = net impulse. PF = peak force. PV = peak velocity. PPa = allometrically scaled peak power: PFa = allometrically scaled peak force. Bold = denotes statistically significant at *p* < 0.05.

**Table 6:** Correlation between initial muscle strength and changes in physical performance.

	B−T1			B−T2			T1−T2		
Variables	IPF	RFD250	NIPF	IPF	RFD250	NIPF	IPF	RFD250	NIPF
SJ0									
JH	0.13 (0.63)	−0.05 (0.85)	0.40 (0.11)	0.23 (0.37)	0.05 (0.86)	**0.50 (0.04)**	0.06 (0.81)	0.09 (0.74)	0.00 (0.99)
NI	0.21 (0.41)	0.12 (0.64)	−0.09 (0.74)	0.12 (0.65)	0.14 (0.59)	−0.30 (0.25)	−0.05 (0.84)	0.06 (0.81)	−0.35 (0.17)
PP	0.22 (0.40)	0.15 (0.56)	−0.06 (0.82)	−0.01 (0.98)	0.05 (0.86)	−0.31 (0.23)	−0.23 (0.38)	−0.10 (0.70)	−0.37 (0.14)
PF	−0.19 (0.46)	−0.02 (0.93)	−0.15 (0.58)	−0.49 (0.05)	−0.21 (0.41)	−0.34 (0.18)	−0.46 (0.07)	−0.25 (0.34)	−0.32 (0.21)
PV	0.38 (0.14)	0.23 (0.38)	0.05 (0.85)	0.24 (0.36)	0.15 (0.56)	−0.18 (0.48)	−0.09 (0.73)	−0.05 (0.84)	−0.31 (0.23)
PPa	0.28 (0.27)	0.16 (0.54)	0.02 (0.94)	0.08 (0.75)	0.09 (0.73)	−0.24 (0.35)	−0.18 (0.48)	−0.06 (0.81)	−0.35 (0.17)
PFa	0.09 (0.70)	0.17 (0.50)	0.01 (0.98)	−0.06 (0.81)	0.21 (0.41)	−0.22 (0.40)	−0.14 (0.59)	0.15 (0.58)	−0.28 (0.27)
SJ20									
JH	0.02 (0.95)	−0.12 (0.65)	0.24 (0.36)	−0.26 (0.31)	−0.23 (0.38)	0.22 (0.49)	−0.27 (0.29)	−0.06 (0.81)	−0.10 (0.69)
NI	0.04 (0.87)	0.05 (0.86)	−0.03 (0.89)	−0.26 (0.32)	0.07 (0.79)	−0.18 (0.49)	−0.36 (0.15)	0.01 (0.98)	−0.18 (0.49)
PP	−0.09 (0.73)	−0.05 (0.84)	0.09 (0.73)	**−0.49 (0.04)**	−0.12 (0.65)	−0.29 (0.26)	−0.35 (0.16)	−0.05 (0.84)	−0.34 (0.18)
PF	−0.29 (0.26)	−0.18 (0.49)	0.02 (0.93)	**−0.52 (0.03)**	−0.27 (0.29)	−0.30 (0.24)	−0.46 (0.06)	−0.20 (0.44)	**−0.51 (0.04)**
PV	0.07 (0.80)	0.06 (0.82)	−0.15 (0.57)	−0.21 (0.41)	0.01 (0.97)	−0.11 (0.67)	−0.32 (0.21)	−0.08 (0.75)	0.06 (0.83)
PPa	−0.02 (0.94)	−0.01 (0.97)	−0.20 (0.44)	**−0.50 (0.04)**	−0.19 (0.46)	−0.24 (0.35)	−0.46 (0.06)	−0.16 (0.55)	−0.04 (0.86)
PFa	−0.21 (0.43)	−0.04 (0.87)	−0.14 (0.59)	**−0.50 (0.04)**	−0.16 (0.52)	−0.31 (0.22)	**−0.59 (0.01)**	−0.22 (0.62)	−0.36 (0.16)
SJ40									
JH	−0.26 (0.32)	−0.26 (0.32)	0.29 (0.26)	**−0.53 (0.03)**	−0.40 (0.12)	0.00 (1.00)	−0.20 (0.44)	−0.06 (0.81)	−0.39 (0.13)
NI	0.23 (0.37)	−0.13 (0.61)	0.37 (0.15)	0.32 (0.21)	0.23 (0.37)	0.14 (0.60)	0.09 (0.72)	0.35 (0.17)	−0.24 (0.36)
PP	0.20 (0.44)	−0.15 (0.54)	0.31 (0.22)	0.26 (0.32)	0.19 (0.46)	0.08 (0.75)	0.04 (0.04)	0.30 (0.24)	−0.25 (0.34)
PF	−0.25 (0.33)	−0.10 (0.70)	0.07 (0.80)	−0.41 (0.10)	−0.21 (0.42)	−0.22 (0.39)	−0.38 (0.13)	−0.24 (0.36)	−0.46 (0.06)
PV	0.26 (0.31)	−0.13 (0.61)	0.29 (0.26)	0.35 (0.17)	0.22 (0.39)	0.14 (0.59)	0.09 (0.72)	0.33 (0.19)	−0.15 (0.56)
PPa	0.22 (0.24)	−0.14 (0.59)	0.28 (0.27)	0.25 (0.35)	0.13 (0.62)	0.09 (0.72)	0.00 (0.99)	0.22 (0.39)	−0.20 (0.43)
PFa	−0.08 (0.77)	−0.13 (0.61)	0.11 (0.67)	−0.19 (0.46)	0.01 (0.98)	−0.20 (0.45)	−0.15 (0.57)	0.15 (0.59)	−0.38 (0.14)
Sprint									
10 m	0.19 (0.47)	**0.48 (0.05)**	−0.18 (0.49)	0.06 (0.82)	0.37 (0.15)	−0.44 (0.08)	−0.14 (0.59)	−0.12 (0.66)	−0.38 (0.14)
20 m	0.36 (0.16)	0.33 (0.20)	−0.06 (0.81)	0.32 (0.21)	0.33 (0.19)	**−0.54 (0.03)**	−0.01 (0.96)	0.03 (0.90)	**−0.59 (0.01)**

Note: values are expressed as correlation coefficient (*p*−value). B = baseline. T1 = the start of the second training block. T2 = the end of the second training block. IPF = isometric peak force. RFD250= rate of force development at 250 milliseconds. NIPF = net impulse to isometric peak force. SJ = static vertical jump. JH = jump height. PP = peak power. NI = net impulse. PF = peak force. PV = peak velocity. PPa = allometrically scaled peak power. PFa = allometrically scaled peak force. Bold = denotes statistically significant at *p* < 0.05.
